# Supplementary material for: Mutual potentiation drives synergy between trimethoprim and sulfamethoxazole
Source: Nat Commun. 2018 Mar 8;9:1003. doi: 10.1038/s41467-018-03447-x (PMC5843663; doi:10.1038/s41467-018-03447-x)
Supplement: Supplementary file 1 — Supplementary Information [file 41467_2018_3447_MOESM1_ESM.pdf]

Mutual Potentiation Drives Synergy between Trimethoprim and Sulfamethoxazole

Minato et al.

a

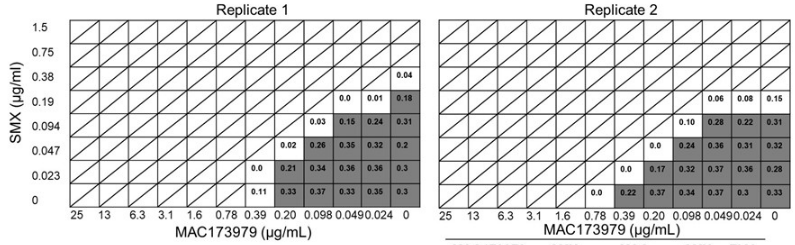

MAC173979 MIC (µg/mL)	SMX MIC (µg/mL)	MAC173979 FIC	SMX FIC	FICI
0.39	0	1.0	0	1.0
0.20	0.047	0.50	0.13	0.63
0.098	0.094	0.25	0.25	0.5
0.024	0.19	0.063	0.5	0.56
0	0.38	0	1.0	1.0

MAC173979 MIC (µg/mL)	SMX MIC (µg/mL)	MAC 173979 FIC	SMX FIC	FICI
0.78	0	1.0	0	1.0
0.39	0.023	0.50	0.13	0.63
0.20	0.047	0.25	0.25	0.5
0.098	0.094	0.13	0.5	0.63
0.024	0.19	0.031	1.0	1.0
0	0.19	0	1.0	1.0

b

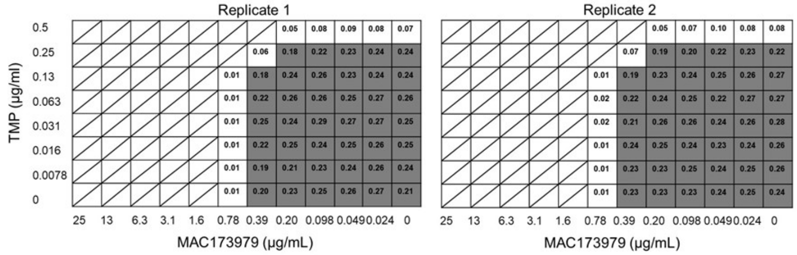

MAC173979 MIC (µg/mL)	TMP MIC (µg/mL)	MAC173979 FIC	TMP FIC	FICI
0	0.5	0	1.0	1.0
0.39	0.25	0.5	0.5	1.0
0.78	0	1.0	1.0	1.0

MAC173979 MIC (µg/mL)	TMP MIC (µg/mL)	MAC173979 FIC	TMP FIC	FICI
0	0.50	0	1.0	1.0
0.39	0.25	0.50	0.50	1.0
0.78	0	1.0	1.0	1.0

c

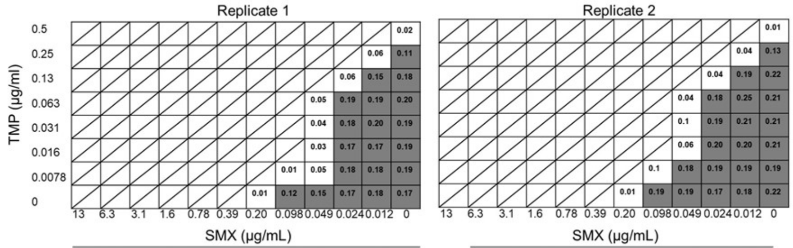

TMP MIC (µg/mL)	SMX MIC (µg/mL)	TMP FIC	SMX FIC	FICI
0.5	0	1	0	1
0.25	0.012	0.50	0.063	0.56
0.13	0.024	0.25	0.12	0.37
0.0078	0.049	0.016	0.25	0.27
0	0.20	0	1	1

TMP MIC (µg/mL)	SMX MIC (µg/mL)	TMP FIC	SMX FIC	FICI
0.5	0	1	0	1
0.25	0.012	0.50	0.063	0.56
0.13	0.024	0.25	0.12	0.37
0.031	0.049	0.063	0.25	0.31
0.0078	0.098	0.016	0.5	0.52
0	0.2	0	1	1.0

Supplementary Figure 1. Inhibitory activity of combinations of anti-folate compounds. *E. coli* BW25113 strain was grown overnight in LB medium. Cultures were washed twice and resuspended in M9-glucose, then inoculated into a 96 well flat bottom plate (Corning) containing the same medium with combinations of a range of concentrations of (**A**) SMX and MAC173979, (**B**) TMP and MAC173979, or (**C**) TMP and SMX. MICs were read spectrophotometrically (OD_{600}) to determine the minimum amount of antimicrobial agent required to inhibit at least 50% of growth relative to a no drug control after 24 hr incubation at 37 °C. Concentration ranges were; SMX (0.023 - 13 µg/ml), TMP (0.0078-0.5 µg/ml), MAC173979 (0.024 - 25 µg/ml). MICs were

determined by visible growth after 24 hr incubation at 37 °C. Synergy was assessed by calculating FICI. Data represents two independent replicates.

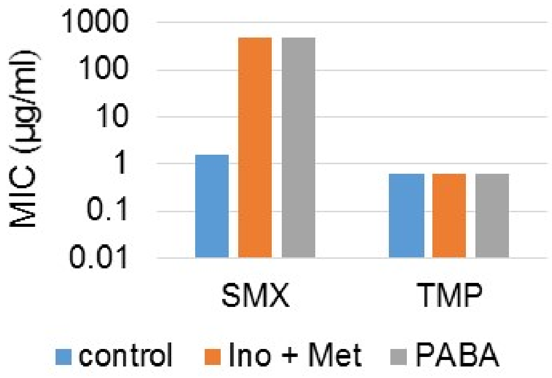

Supplementary Figure 2. Effects of inosine (Ino) and methionine (Met), PABA on SMX and TMP MICs against *E.coli* BW25113. Ino, Met, and PABA were added to the media at 10 µg/ml. MICs were determined after 24 hour incubation at 37 °C in M9-glucose media. Representative data from at least three individual experiments are shown.

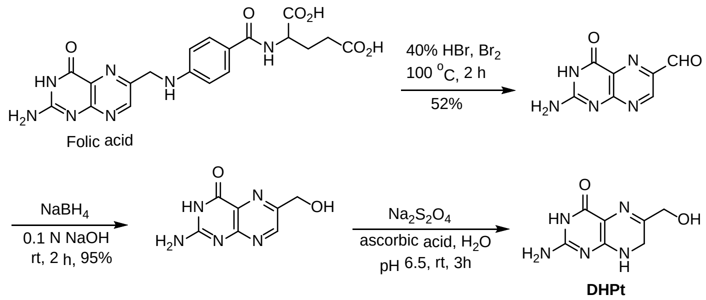

Supplementary Figure 3. Synthesis of DHPt.

Supplementary Table 1. MICs of ceftazidime and ciprofloxacin against *E. coli* BW25113 strains.

Strains	MIC (µg/ml)	
	Ceftazidime	Ciprofloxacin
BW25113 (wild type)	0.02-0.04	0.005-0.01
Δ*nudB*	0.02-0.04	0.005-0.01

MIC, Minimum concentration of antimicrobial agent required to inhibit at least 50% of growth relative to a no drug control after 24 hours of incubation at 37 °C

Supplementary Table 2. MICs of SMX and TMP against *E.coli* BW25113 strains.

Strains	MIC (µg/ml)	
	SMX	TMP
BW25113 (wild type)	1.6	0.6
Δ*nudB*/pUC19-*nudB*	1.6	0.6
Δ*gcvP*/pUC19-*gcvP*	1.6	0.6
Δ*gcvH*/pUC19-*gcvH*	1.6	0.6
Δ*gcvT*/pUC19-*gcvT*	1.6	0.6

MIC, Minimum concentration of antimicrobial agent required to inhibit at least 50% of growth relative to a no drug control after 24 hours of incubation at 37 °C; SMX, sulfamethoxazole; TMP, trimethoprim

Supplementary Table 3. Plasmids and bacterial strains used in this study

Strains/plasmid	Descriptions	Source or reference
WT	*E.coli* BW25113	Keio collection [1]
Δ*pabC*	BW25113 *pabC*::km, kanamycin resistant (Km^r)	Keio collection [1]
Δ*nudB*	BW25113 *nudB*::km, Km^r	Keio collection [1]
Δ*glyA*	BW25113 *glyA*::km, Km^r	Keio collection [1]
Δ*gcvP*	BW25113 *gcvP*::km, Km^r	Keio collection [1]
Δ*gcvH*	BW25113 *gcvH*::km, Km^r	Keio collection [1]
Δ*gcvT*	BW25113 *gcvT*::km, Km^r	Keio collection [1]
Δ*ygfA*	BW25113 *ygfA*::km, Km^r	Keio collection [1]
E. coli B11	Clinical isolate of *E. coli*	Betsy Hirsch[2]
S. aureus USA300	methicillin-resistant *Staphylococcus aureus* strain	[3]
pUC19	Cloning vector, Penicillin resistant (Pen^r)	[4]
pUC19-*nudB*	*nudB* cloned into pUC19, Pen^r	This study
pUC19-*gcvP*	*gcvP* cloned into pUC19, Pen^r	This study
pUC19-*gcvH*	*gcvH* cloned into pUC19, Pen^r	This study
pUC19-*gcvT*	*gcvT* cloned into pUC19, Pen^r	This study

Supplementary Table 4. Primers used in this study.

Primer	Sequence (5' to 3')
Fw_ECnudB_BamHI	CGGGATCCGTGAAGGATA AAGTGTATAA
Re_ECnudB_EcoRI	CGGAATTCTCAGGCAGCGTTAATTACAA
Fw_ECgcvP_BamHI	CGGGATCCATGACACAGA CGTTAAGCCA
Re_ECgcvP_EcoRI	CGGAATTCTTACTGGTATTCGCTAATCGG
Fw_ECgcvH_BamHI	CGGGATCCATGAGCAACG TACCAGCAGA
Re_ECgcvH_EcoRI	CGGAATTCTTACTCGTCTTCTAACAATG
Fw_ECgcvT_BamHI	CGGGATCCATGGCACAAC AGACTCCTTT
Re_ECgcvT_EcoRI	CGGAATTCTCACGCGACGGCTTTGCCGTTA

Supplementary Table 5. Key fragmentation and optimized mass spectrometer (QTRAP 5500) conditions.

Compound	Precursor ion	Product ion	CE (V)
DHPt	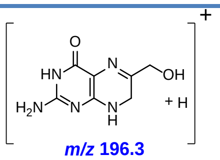 ***m/z* 196.3**	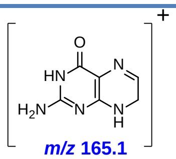 ***m/z* 165.1**	27.0
6-Hydroxymethyl pterin	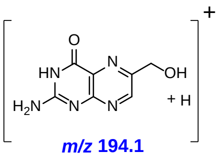 ***m/z* 194.1**	$[M\text{-}H_2O]^+$ ***m/z* 176.1**	27.0
DHN	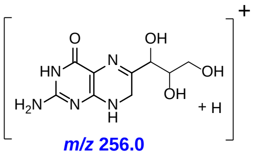 ***m/z* 256.0**	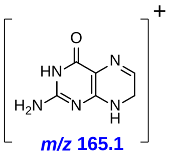 ***m/z* 165.1**	25.0
Neopterin	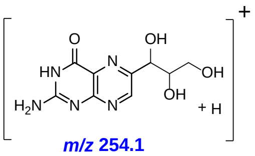 ***m/z* 254.1**	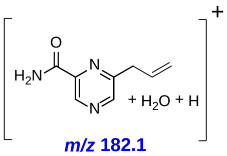 ***m/z* 182.1**	25.0
Hydroxypetroic acid	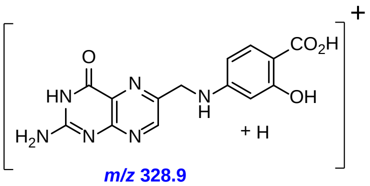 ***m/z* 328.9** **Internal Standard**	$[M\text{-}H_2O]^+$ ***m/z* 311.0**	20.0
PABA	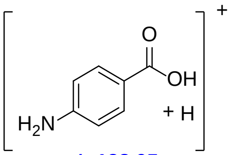 ***m/z* 138.05**	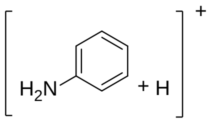 ***m/z* 94.07**	20.0
d_4-PABA	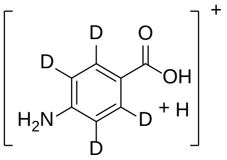 ***m/z* 142.08** **Internal standard**	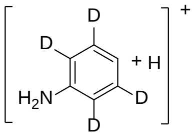 ***m/z* 98.09**	20.0

CE = Collision Energy; Declustering Potential (DP) = 35.0 V, Entrance Potential (EP) = 10.0 V, Collision Cell Exit Potential (CXP) = 15.0 V were kept constant for all transitions.

References.

1 Baba, T. *et al.* Construction of Escherichia coli K-12 in-frame, single-gene knockout mutants: the Keio collection. *Mol Syst Biol* **2**, 2006.0008, doi:10.1038/msb4100050 (2006).

2 Hirsch, E. B. *et al.* Activity of fosfomycin and comparison of several susceptibility testing methods against contemporary urine isolates. *Int J Antimicrob Agents* **46**, 642-647, doi:10.1016/j.ijantimicag.2015.08.012 (2015).

3 Kennedy, A. D. *et al.* Epidemic community-associated methicillin-resistant Staphylococcus aureus: recent clonal expansion and diversification. *Proc Natl Acad Sci U S A* **105**, 1327-1332, doi:10.1073/pnas.0710217105 (2008).

4 Yanisch-Perron, C., Vieira, J. & Messing, J. Improved M13 phage cloning vectors and host strains: nucleotide sequences of the M13mp18 and pUC19 vectors. *Gene* **33**, 103-119 (1985).
